# The Routine Treatment of Fresh Gonorrhœa and Straight Homœopathy

**Published:** 1890-11

**Authors:** Thomas Skinner

**Affiliations:** London, England


### "THE ROUTINE TREATMENT OF FRESH GONORRHŒA AND *STRAIGHT* HOMŒOPATHY."

THOMAS SKINNER, M. D., LONDON, ENGLAND.

As an illustration of the evil effects of urethral injections in cases of fresh infection of gonorrhœa, advocated by Professor Timothy F. Allen, I give the following case, and most men in large practice and of long experience must have seen any number of much the same, and many more infinitely worse, treated by old-school practitioners.

I was consulted on the 9th of July, 1890, by a gay bachelor of forty-five summers, who had been treated by "a routine *straight* homœopathic physician," not a thousand miles from the West End of London, and one who enjoys a large share of public confidence.

The gentleman complained to me of losing the sight of both eyes, the sight of the left being the most impaired. The conjunctiva was inflamed and secreting hot tears, there was an unpleasant sensation of coarse sand in the left eye, with a somewhat muco-purulent discharge. At the same time he complained of the right testicle being painfully enlarged.

I at once asked him if he had had a gonorrhœal discharge? To which he replied: "Oh! yes, but it has been *cured* by Dr. ——, one of your sort, a homœopath." "When did he cure you?" "About a week ago, and it was a fresh infection caught only a few nights before."

The *modus medendi* was the Iodide of Mercury in a low power internally, three times a day, and *Hydrastis in water as a urethral injection, to be repeated thrice daily. Hinc illœ lachrymœ.*

Here was a nice little specimen of Dr. Timothy Allen's "straight Homœopathy." Will he be kind enough to quote from *The Organon* of Hahnemann or from *any* of Samuel Hahnemann's writings, that the master ever advised the use of local injections for the cure of any discharge, especially an infectious one, containing a virulent poison?

TREATMENT.—The medicines best indicated seemed to me Sulphur and Pulsatilla, and inasmuch as the latter corresponds best to the swelled testicle, and to the eye, the form of the inflammation assuming that of a pterygium, I gave him on July 9th, 1890, *Puls.*$^{1m}$ (F. C.) to be taken three times a day. He called a week after, and reported all pain in testicle gone, but he continues to wear a suspender, which he has worn from the first appearance of the discharge. The testicle remains as much swollen as before, and the ophthalmia (gonorrhœal) is decidedly better, but at a standstill.

There being no return of the discharge, I gave my patient then and there a dose of *Medorrhinum*$^{cm}$ (Swan) on his tongue, and one more to be repeated at bedtime, and to follow both up with S. L. night and morning. As my patient was called on business of importance to the Continent, he got enough S. L. to last him until his return on the 8th of August last, when he reported himself marvelously better as regards the swollen testicle —and no wonder—*as the urethral discharge of a yellowish-green color had returned*, and the ophthalmia was if anything worse. He now got one dose at bedtime of *Medorrhinum*$^{3mm}$ (Swan) to be followed by S. L. every night. On the 10th of September, the ophthalmia was gone aud the indistinctness of vision; the testicle almost its normal size and feel; and there was nothing to prescribe for except the return of the suppressed gonorrhœa, for which I prescribed, as it was a yellow discharge of apparently a healthy pus, *Mercurius solubilis*$^{1m}$, night and morning.

September 17th, 1890.—My patient considers himself now quite well, and expressed himself deeply grateful, because "Doctor, I felt certain that I was *poisoned!*"

With all deference to Dr. Timothy Allen—and be it remembered he is a "teacher in Israel" and a great power for good or evil in the land—he is teaching that which every honest and advanced allopath condemns, namely, the suppression of infectious poisons by means of injections, such as gonorrhœa.

Were it not for the shame of the thing, I wonder that patients do not come upon such "straight homœopaths," eclectics, and *allopaths*, and sue them at law for malpractice, for the suppression of virulent infectious poisons and inducing orchitis, ophthalmia, rheumatism, etc., in their persons; whereas, they were paid for *curing* them of a running, *and not for shutting up the burglar in the house.*

Let us hope that Professor Timothy Allen, M. D., will change his *methodus medendi* of treating acute gonorrhœal inflammation of the male urethra, and that we shall hear no more of such "straight Homœopathy," as "He who in these days will not *wash out* with distilled water and one 5,000th of a grain of Corrosive Mercury a fresh case of gonorrhœa, and cure (*sic*) his erring brother in twenty-four or forty-eight hours, must give up the treatment of such diseases." Know this, then, Dr. Allen, that we can cure effectively all cases of gonorrhœa *cito, tuto, et jucunde*, without *local* treatment of any kind, not even the injection of the 5,000th of a grain of Corrosive Sublimate—which cannot "wash out" the poison except by neutralizing or suppressing it. *My patient* told me he was *cured by a homœopath*, when he was simply made dangerously ill—and if any one has seen or is able to trace the evil effects of these *cures* by suppression, they would as soon think of cutting off their own right hand or of killing their patient.

